# Modes of Attention and the Clinical Mind: Distinguishing Meditation, Mindfulness, and Contemplation in Psychiatry

**DOI:** 10.1192/j.eurpsy.2026.11894

**Published:** 2026-07-31

**Authors:** J. B. French, J. Kar, B. R. Carr

**Affiliations:** 1 University of Florida College of Medicine, Gainesville, FL, United States; 2 Department of Psychiatry, University of Florida College of Medicine, Gainesville, FL, United States

## Abstract

**Introduction:**

The psychiatric incorporation of mindfulness has advanced without a parallel precision in definition. Within both clinical and lay discourse, mindfulness is often conflated with meditation broadly, or with forms of contemplation oriented toward ethical or spiritual ends. This conceptual slippage obscures distinct phenomenological structures and risks importing unexamined assumptions into psychiatric care. Drawing from philosophy of mind, phenomenology, and clinical literature, this project examines the attentional, affective, and epistemic contours that differentiate meditation, mindfulness, and contemplation, and their respective implications for psychiatric practice.

**Objectives:**

To (1) differentiate meditation, mindfulness, and contemplation as distinct attentional modes; (2) map their cognitive-affective mechanisms and therapeutic relevance; and (3) identify ethical and clinical consequences of categorical conflation.

**Methods:**

A targeted literature review was conducted across psychiatry, cognitive neuroscience, and philosophical sources on attention, self-regulation, and contemplative traditions. Reference mining from seminal works supplemented database searches. Analytical comparison was guided by phenomenological description and conceptual analysis, with emphasis on constructs applicable to psychiatric intervention.

**Results:**

Meditation is best understood as a structured practice of sustained attentional modulation, which may include but is not limited to mindfulness. Mindfulness, when defined with clinical precision, constitutes a present-focused, non-reactive attentional stance aimed at reducing maladaptive cognitive elaboration. Contemplation, in contrast, privileges intentional reflection—often value-laden—toward moral, spiritual, or existential objects. These modes diverge in intentionality, cognitive load, and affective orientation, with empirical evidence supporting mindfulness as the most directly applicable to relapse prevention in depression and anxiety (Psychother Psychosom 2018;87:184-186). Conflating these constructs risks inappropriate application, ethical overreach, and dilution of therapeutic effect.

**Conclusions:**

A philosophy of psychiatry approach clarifies that the clinical import of mindfulness depends on its distinction from related practices. When these attentional modes are properly differentiated, psychiatry can deploy mindfulness with greater epistemic transparency, cultural sensitivity, and therapeutic precision—avoiding the erosion of its conceptual integrity under the weight of undifferentiated wellness rhetoric.

**Disclosure of Interest:**

None Declared

